# Renal Artery Stenting for Traumatic Dissection

**DOI:** 10.7759/cureus.95413

**Published:** 2025-10-25

**Authors:** Glenn Yang Han Ng, Joel Jingkai Liu, Haiyuan Shi, Kentson Jing Xin Lee

**Affiliations:** 1 Radiology, Changi General Hospital, Singapore, SGP

**Keywords:** acute renal injury, arterial dissection, renal artery stenting, renal involvement, renovascular hypertension

## Abstract

Renal injuries are frequently seen in the context of trauma, with currently well-established guidelines for classification and management, such as those prescribed by the American Association for the Surgery of Trauma. However, traumatic dissection of the renal arteries specifically is uncommon; clarity regarding optimal management is currently lacking, despite the possible long-term sequelae, including loss of nephron function and renovascular hypertension. While medical management alone had sometimes been suggested, endovascular stenting is potentially a viable minimally invasive intervention that may further reduce the risk of long-term complications. In this report, we present a case of traumatic renal artery dissection first seen on computed tomography in a patient who presented following a high-speed road traffic accident. Percutaneous endovascular stenting of a dissected renal artery was performed, with subsequent restoration of renal perfusion on follow-up imaging, as well as maintenance of normal serum creatinine and blood pressure at longer-term follow-up. We also review the existing literature, which, despite the scarcity, affirms our experience of favourable outcomes post-intervention.

## Introduction

Renal artery dissection is rare, with spontaneous renal artery dissections forming just 1-2% of all arterial dissections [1]. Traumatic causes are even less common, accounting for 12% of renal arterial dissections [2]. Dissection of the renal artery with flow limitation results in acute renal infarction, which, if left untreated, may result in permanent impairment of renal function [3] and/or renovascular hypertension [4]. Early recognition and management are of paramount importance in mitigating the risks of such.

Although detection of renal artery dissection following trauma is increasing due to the routine use of contrast-enhanced computed tomography (CT), controversy over ideal management exists due to the current lack of firmly established guidelines [5]. For instance, while the World Society of Emergency Surgery and American Association for the Surgery of Trauma (WSES-AAST) guidelines for kidney and urogenital trauma [6] do state that stenting is "indicated" for haemodynamically stable patients with severe renal trauma and main renal artery dissection, it also labels this as a level 2C recommendation (weak recommendation with low-quality evidence) with no further explanation offered.

In this article, we present a case of traumatic renal artery dissection with evidence of renal hypoperfusion on initial CT, for which percutaneous endovascular stenting was performed, with restoration of renal perfusion seen on subsequent follow-up imaging. We also explore the current state of the literature relevant to this topic and discuss the merits of stenting in the context of traumatic renal artery dissection.

## Case presentation

A previously healthy 49-year-old gentleman presented to the hospital following a high-speed road traffic accident; he was unconscious on arrival, and further history could not be obtained. On arrival to the emergency department, his blood pressure was 145/79, and his heart rate was 101 per minute. Serum creatinine level was 90 µmol/L.

Following fluid resuscitation and achievement of haemodynamic stability, a whole-body CT was performed as per our institution’s trauma protocol; this included early arterial phase imaging of the chest, abdomen, and pelvis, followed by portal venous and delayed phases of the abdomen and pelvis. Our scans were performed at 120 kVp and 120 mAs; following contrast injection, the arterial, portal venous, and delayed phases were acquired at 15 seconds, one minute and 10 seconds, and four minutes and 20 seconds, respectively, with a slice thickness of 3.0 mm on all phases.

**Figure 1:**
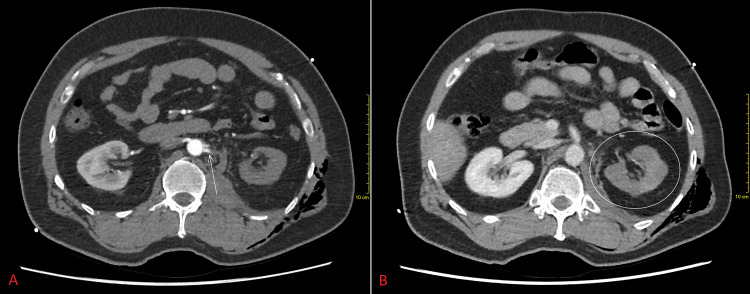
Abrupt truncation of the left renal arteries (panel A, arrow), with faint patchy renal enhancement on the delayed phase (panel B, circle). Hounsfield units of the kidneys (cortex/medulla) were as follows: 117/74 on the right versus 35/33 on the left in the early arterial phase; 185/118 on the right versus 71/36 on the left in the portal venous phase. The arterial stump measured 234 Hounsfield units. In this figure, the window widths/centres were respectively 500/70 for the early arterial phase and 400/40 for the portal venous phase.

Left renal hypoenhancement was seen on the arterial phase with abrupt truncation of the left renal arteries proximally; faint patchy enhancement was noted on the delayed phase (Figure 1). There was no contrast extravasation or venous pooling to suggest active haemorrhage or an arterial rupture. Other injuries seen on the CT included left-sided rib fractures with haemopneumothorax and subcutaneous emphysema, left adrenal haematoma, left T12 to L3 and right L4 to L5 transverse process fractures.

The urology team was consulted; given the renal devascularisation without active haemorrhage, this was classified as an AAST Grade IV renal injury. The patient was then referred to us for stenting of the left renal artery due to evidence of flow limitation on CT.

The procedure commenced 3.5 hours after the estimated time of the accident; the patient’s blood pressure was measured at 112/78. Ultrasound-guided access of the right common femoral artery was obtained via a 5 Fr sheath. Through this, the left renal arteries (main renal artery and lower pole accessory renal artery) were cannulated with a 5 Fr, and angiography was then performed. This showed that both arteries were occluded near their origins, likely secondary to traumatic dissections and thrombosis, with sparing of the major renal hilar branches. We decided to proceed with the treatment, aiming to reperfuse the majority of the left kidney via the left main renal artery.

**Figure 2:**
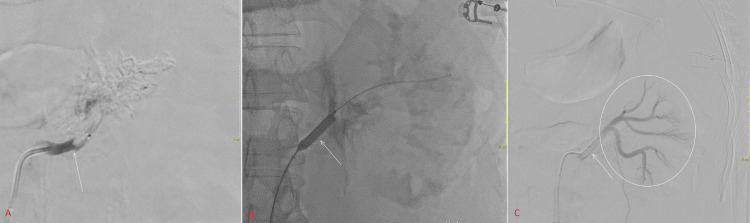
Demonstration of dissection (arrow, panel A), stent deployment (arrow, panel B) and post-stent angiography showing the stent in-situ (arrow, panel C) with good distal flow (circle).

Intraluminal crossing of the left main artery occluded segment with a 2 Fr microcatheter (TruSelect; Boston Scientific, Marlborough, United States) and 0.014-inch microwire (ASAHI Meister S14; Asahi Intecc, Seto, Japan) was achieved after a few attempts. Angiography past the occlusion showed good perfusion of the distal branches. Over a stiff wire (V-18 ControlWire; Boston Scientific, Marlborough, United States), a 6 Fr guide sheath was advanced past the dissection. The diameter of the left main renal artery was 5 mm, the distance of the dissection from the ostium was 7 mm, and the involved segment was approximately 15 mm in length. We then deployed a 6 x 22 mm balloon-mounted stent (BeGraft Peripheral; Bentley InnoMed, Hechingen, Germany); the landing zone was proximally at 4 mm from the ostium and distally at 3 mm past the involved segment. The stent was sited 9 mm from the renal bifurcation; this was done to prevent occlusion of the major branches arising from the renal hilum. Final angiography showed good flow through the main renal artery with preservation of major branches (Figure 2). Some flow was noted through the accessory renal artery, but the decision was made not to pursue further intervention.

No intra-procedural anticoagulation was given. The procedure was performed with true anteroposterior projection throughout. Approximately 150 ml of Omnipaque 350 was administered. The total fluoroscopy time was 2427 seconds.

The blood pressure post-procedure was 114/88. To ensure stent patency, the patient was subsequently started on permanent single antiplatelet therapy (100 mg of aspirin daily); while initial short-term dual antiplatelet therapy was briefly considered, the clinical team opted for the former due to concerns of hemorrhage, given the multiple other concomitant injuries as mentioned.

**Figure 3:**
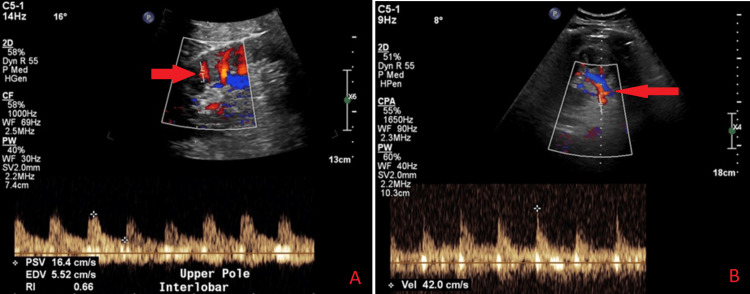
Post-stent Doppler ultrasound examination of the left kidney shows preserved perfusion (panel A), as well as the patent distal left main renal artery with a normal peak systolic velocity (panel B). The interlobar arteries showed a normal peak PSV, EDV and RI of 16.4 cm/s, 5.52 cm/s, and 0.66 in the upper pole; 19.4 cm/s, 6.6 cm/s, and 0.66 in the middle pole; and 24.6 cm/s, 8.46 cm/s, and 0.66 in the lower pole, respectively. The visualised distal segment of the main left renal artery was patent with a normal peak systolic velocity of 43.1 cm/s.

Doppler ultrasound of the left kidney three days post-stenting showed good left renal perfusion; the interlobar arteries showed normal peak systolic velocities (PSV), end-diastolic velocities (EDV), and resistive indices (RI). The proximal and middle segments of the left main renal artery and the stent were not well-visualised, but the distal segment was patent with normal peak systolic velocity (Figure 3). No tardus et parvus waveform was observed. The patient’s serum creatinine, which was initially elevated one day post-procedure (127 µmol/L), eventually normalised within 10 days (81 µmol/L).

**Figure 4:**
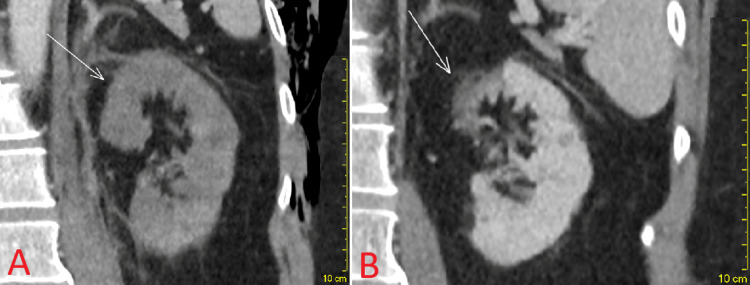
Appearances on the left kidney on the initial CT (panel A) and follow-up 11 months post-discharge (panel B). Note that the maximum cortical thickness in the upper pole decreased from 1.7 to 1.0 cm (arrows).

At the outpatient follow-up 10 months post-discharge, repeat Doppler ultrasound showed good renal perfusion with normal PSV, EDV, and RI of 24.6 cm/s, 8.80 cm/s, and 0.64 in the upper pole; 18.8 cm/s, 6.4 cm/s, and 0.66 in the middle pole; and 25.5 cm/s, 10.2 cm/s, and 0.66 in the lower pole, respectively. The visualised distal and middle segments of the left renal artery demonstrated normal velocities of 33.2 cm/s and 62.5 cm/s, respectively. Again, no tardus et parvus waveform was seen. CT performed 11 months post-discharge showed decreased left kidney length from 9.8 cm to 8.3 cm with cortical scarring (Figure 4). 

He reported being asymptomatic and did not develop hypertension according to clinical documentation, and the serum creatinine remained within normal limits (103 µmol/L).

## Discussion

Traumatic renal artery dissection is rare; a review of the National Trauma Data Bank by Sangthong et al revealed that renal artery injuries were only documented in 0.05% of blunt trauma admissions [7]. This probably contributes to the current absence of established protocols.

Systematic review and meta-analysis by Petrone et al. on the literature regarding traumatic renal injuries show that conservative management is frequently adequate, even in patients with high AAST grade injuries, provided that they are haemodynamically stable [8]. Furthermore, endovascular intervention, while minimally invasive, is not completely risk-free. For example, Rozzaingo et al. described a patient who had developed intra-procedural hypotension as a result of iatrogenic arterio-calyceal fistulation during renal angiography [5]. Therefore, anticoagulation alone had been the mainstay of treatment, especially in cases of dissection with otherwise no flow limitation [2,9].

However, for our patient with CT-proven flow limitation and an occlusive flap location proximal to the hilum, we elected to perform endovascular stenting to attempt renal revascularisation. Despite the "weak recommendation" label previously mentioned, the WSES-AAST guidelines do suggest that stenting is indicated for our patient who was haemodynamically stable, had severe renal trauma (AAST Grade IV), and had main renal artery dissection with limited warm ischaemia time, which the guidelines classified as <240 minutes [6]. Furthermore, nephron preservation was of interest in our relatively healthy patient with good pre-morbid function; given the susceptibility of the kidneys to warm ischaemia, with complete occlusion of the renal artery leading to worse outcomes due to paucity of collateral flow beyond the point of occlusion, revascularisation is thought to be of paramount importance in preservation of renal function [10]. Despite the scarcity, currently available case reports show that renal artery stenting in adult patients with traumatic dissections leads to preserved renal function in the long term [5,10,11]. Although normal biochemistry results can potentially be a misleading indicator of preserved renal function in a patient with a normal contralateral kidney [12], the cases reported by Abu-Gazala et al. were also followed up with radionuclide perfusion scans, which showed relative renal functions of 49% and 40% in their two adult patients who received endovascular stents [10]. Taking 25% as the definition of renal salvage [13], this study affirms the efficacy of this technique in nephron preservation. Our patient had normal serum creatinine one year post-discharge; despite the previously mentioned limitation of biochemistry results in determining renal function, our outcomes remain consistent with those of previously reported cases. 

In addition, renovascular hypertension is a known complication of traumatic renal artery injury; this is due to the activation of the renin-angiotensin system in response to poor renal perfusion [14]. Although a systematic review of 129 case reports by Jha et al. found that optimal medical management alone improved hypertension in 90% of cases [2], many of these studies involved spontaneous renal artery dissections, rather than traumatic dissections. Furthermore, persistent renovascular hypertension post-trauma is the main reason for eventual nephrectomy [15]. Our patient was not hypertensive prior to stenting. However, trauma-related renovascular hypertension can present weeks to months after the injury [15]; the European Association of Urology guidelines therefore suggest monitoring for renovascular hypertension for at least one year post-trauma [16]. Taking these into account, the outcomes of our case suggest that early renovascular intervention, despite the initial absence of hypertension, can be beneficial in preventing future late-onset hypertension and, in turn, eventual nephrectomy.

## Conclusions

This case highlights that in the setting of CT-demonstrated flow limitation from traumatic renal artery dissection, early endovascular stenting is a reasonable first-line option to consider for nephron salvage in appropriately selected, haemodynamically stable patients. While the broader literature remains small and heterogeneous, our outcome was consistent with prior reports but not definitive. Further studies and pooled data will be essential to establish standardised guidelines, but current evidence, including our case, strongly advocates for stenting as a viable and effective management strategy in this context.
